# Clinical and socio-demographic predictors for virologic failure in rural Southern Africa: preliminary findings from CART-1

**DOI:** 10.7448/IAS.17.4.19666

**Published:** 2014-11-02

**Authors:** Niklaus Daniel Labhardt, Joëlle Bader, Mojakisane Ramoeletsi, Mashaete Kamele, Thabo Ismael Lejone, Molisana Cheleboi, Mokete M. Motlatsi, Jochen Ehmer, Olatunbosun Faturyiele, Daniel Puga, Thomas Klimkait

**Affiliations:** 1 Department of Medical and Diagnostic Services, Swiss Tropical and Public Health Institute, Basel, Switzerland; 2 Department Biomedicine, University of Basel, Basel, Switzerland; 3 Laboratory Services, Paray Hospital, Thaba-Tseka, Lesotho; 4 SolidarMed, SolidarMed Lesotho, Thaba-Tseka, Lesotho; 5 Laboratory Services, Seboche Hospital, Botha-Bothe, Lesotho; 6 SolidarMed, SolidarMed Switzerland, Lucerne, Switzerland; 7 SolidarMed, SolidarMed Lesotho, Botha-Bothe, Lesotho

## Abstract

**Introduction:**

In 2013, the World Health Organization (WHO) recommended scaling up of routine viral load (VL) monitoring for patients on antiretroviral therapy (ART) in resource-limited settings [1]. During the transition phase from no VL-testing at all to routine VL-monitoring, targeted VL for groups at particular risk of virologic failure (VF) may be an option [2]. We present socio-demographic and clinical risk factors for VF in a cohort in rural Lesotho with no access to VL prior to the study.

**Materials and Methods:**

Data derive from a cross-sectional study providing multi-disease screening as well as VL testing to adult patients (≥16 years old) on first-line ART ≥6 months [3]. VF was defined as VL≥1000 copies/mL. Assessed potential predictors of VF were: (1) socio-demographic (sex, age, wealth-quintile, education, employment status, disclosure of HIV status to environment, travel-time to facility); (2) treatment history (history of treatment interruption >2 days, previous drug substitution within first-line ART, time on ART, ART-base and -backbone); (3) adherence (pill count) and (4) clinical (clinical or immunological failure as defined by WHO guidelines [1], presence of papular pruritic eruption (PPE)). All variables with association to VF in univariate analysis were included in a multivariate logistic regression reporting adjusted Odds ratios (aOR).

**Results:**

Data from 1,488 patients were analyzed. Overall VF-prevalence was 6.9% (95% CI 5.7–8.3). In univariate analysis, the following were associated with VF: age <30, lower wealth-quintile, no primary education, history of treatment interruption, nevirapine-base, zidovudine-backbone, history of drug substitution, travel-time to clinic ≥2 hours, disclosure of HIV status to <5 persons, clinical failure, presence of PPE and immunological failure. In multivariate analysis, 6 out of the above 12 variables were independent predictors: age <30 years (aOR: 2.4; 95% CI 1.1–5.3, p=0.029), history of treatment interruption (2.5; 1.3–4.7, p=0.005), PPE (6.9; 2.5–18.9, p<0.001), immunological failure (11.5; 5.7–23.2, p<0.001), history of drug substitution (1.9; 1.0–3.7, p=0.043), disclosure of HIV status to <5 persons (1.8; 1.1–3.1, p=0.03).

**Conclusion:**

In this cohort in rural Lesotho, several socio-demographic and clinical predictors were associated with VF. Particularly age <30 years, history of treatment interruption, PPE and immunological failure were strongly associated with VF. These patients may be prioritized for targeted VL-testing.
